# Socioeconomic characteristics, paternal smoking and secondhand tobacco smoke exposure among infants in Jakarta, Indonesia

**DOI:** 10.18332/tid/120077

**Published:** 2020-05-05

**Authors:** Siti R. Nadhiroh, Kusharisupeni Djokosujono, Diah M. Utari

**Affiliations:** 1 Department of Nutrition, Faculty of Public Health, Universitas Airlangga, Surabaya, Indonesia; 2 Department of Nutrition, Faculty of Public Health, Universitas Indonesia, Depok, Indonesia

**Keywords:** paternal smoking, secondhand tobacco smoke, infants, socioeconomic characteristics

## Abstract

**INTRODUCTION:**

Prevalence of paternal smoking is high in Asia and babies are vulnerable to secondhand tobacco smoke (SHS) exposure at home. This study assesses socioeconomic characteristics and paternal smoking in households and infants’ exposure to SHS.

**METHODS:**

A cross-sectional analysis of data collected as part of a prospective cohort study was conducted in Jakarta, Indonesia during 2017–2019. Participants were 156 mother-baby pairs whose babies reached the age of 6 months. Socioeconomic characteristics and smoking behaviour in the household were assessed by questionnaires. Factors related to paternal smoking and infants’ exposure to SHS were assessed using a multivariate logistic regression model.

**RESULTS:**

Almost two-thirds of infants lived with fathers who were smokers. Lower levels of paternal education (OR=2.59; 95% CI: 1.19–5.63; p=0.045) and infants with one sibling (OR=2.41; 95% CI: 1.02–5.67; p=0.044) increased the risk of paternal smoking in the household. Moreover, infants with one sibling (OR=3.09; 95% CI: 1.15–8.32; p=0.026), lower level of father education (OR=18.73; 95% CI: 1.54–227.93; p=0.022), and a high number of other household members who smoke (OR=4.54; 95% CI : 1.42–14.48; p=0.011) were the risk factors of SHS exposure among infants at home.

**CONCLUSIONS:**

These findings demonstrate the significant influence of educational level, number of children and/or number of other smokers in the household on paternal smoking and SHS exposure among infants at home. Comprehensive tobacco control programmes to increase adoption of smoke-free homes are likely to be an effective way to reduce SHS exposure and promote decreased cigarette smoking in families with children.

## INTRODUCTION

Passive smoking, also called secondhand smoke (SHS), is defined as any tobacco smoke exposure as a result of other peoples’ active smoking^1^. More than one-third of the world’s population is exposed to SHS and the harmful effects of regular cigarette smoke^2^. In Indonesia, around 225720 people die annually from tobacco-related disease^3^. However, the number of smokers aged >15 years is about 89.6 million people or 33.8% of the population. Of these, 62.9% are male and 4.8% female smokers^4^, most smoke at home^4,5^.

Indonesia is the only country in Asia which is not a party to the World Health Organization Framework Convention on Tobacco Control (WHO FCTC). It has some of the weakest tobacco control measures in South-East Asia^6^ and although Indonesia implemented MPOWER measures to diminish tobacco use, none of the measures has worked^7^. Therefore, domestic studies on smoking behaviour and SHS exposure among Indonesians are needed as there exist few data on the prevalence of SHS in infants in Indonesia. However, it has been reported that about 57.3% of the children (13–15 years) and 78.4% of the adults are exposed to SHS at home^5,8^. Babies become vulnerable to cigarette smoke exposure because most of their time is spent at home^9^, and this has become a significant public health issue.

The adverse health effects of SHS may include the risk of sudden infant death syndrome (SIDS), birth defects, acute respiratory illnesses and chronic respiratory disease, cancer and other diseases^10–12^, low birthweight^11,13,14^, and shorter body length^15^. Postnatal cigarette smoke exposure also can be a reason for the decrease in weight and length growth in the early months of life^16^. In children, up to eight years of age^17^, SHS has been associated with meningococcal carriage and disease, and medically attended accidents in children^18^. In addition, SHS exposure is associated with an increased risk of poor neurodevelopment^19^.

In the first 1000 days of life, socioeconomic factors may play a role in nutrition-sensitive causes of malnutrition^20^. Infants aged 6 months are introduced to complementary foods. Nutritious foods guarantee growth and health. For people with a lower middle income, setting aside a portion of their budget for their baby’s nutritional needs is necessary. For this reason, if parents smoke, the dietary needs of their children may be excluded or will not be a priority. Among the poor households in the urban slum areas of Indonesia, paternal smoking results in the diversion of household money from food to tobacco, thereby contributing to child malnutrition^21^.

Research on exposure to cigarette smoke among 6-month-old babies via paternal smoking in middle-low-income families is an area of regional importance. The aim of this study is to assess socioeconomic characteristics and paternal smoking in households and infants’ exposure to SHS in Indonesia.

## METHODS

### Study design, setting and participants

The data for this cross-sectional study were derived from an ongoing PEER (Partnerships for Enhanced Engagement in Research) Health prospective cohort study in Jakarta, Indonesia. The primary objective of the cohort study is to determine the effects of individual air pollution exposure in pregnancy and early infancy on maternal health (lung function, hypertension disorder in pregnancy, or preeclampsia) and neonatal/infant health (lung function and infection episodes).

The study population consists of pregnant women attending antenatal care and arranging to deliver the babies in seven community health centers in Jakarta, Indonesia. The study includes all pregnant women in the first antenatal visit, then followed up until the babies reach 6 months of age. Only women who have lived in their residential area for three months or more before the pregnancy are included in the study. Based on the sample size calculations, the study consists of 600 mother-infant pairs, starting from 2016 and finishing in 2020. After enrollment, pregnant mothers are followed up in line with routine antenatal care (ANC). After delivery and discharge, infants are invited for routine postnatal visits at the age of 40 days and at 2, 4 and 6 months. For those who refuse, a separate team of field workers visit the households.

To assess the socioeconomic characteristics and household risk factors related to paternal smoking and SHS exposure in 6-month-old infants, we limited participants to those with infants who reached the age of 6 months. We further included those with completed data for socioeconomic characteristics and smoking in households until July 2019. Thus, the present study includes 156 mother-infant pairs.

### Measures

The questionnaire was developed by the PEER Health international team of experts. The questionnaire was pilot tested with 10 mothers resulting in no changes. Socioeconomic characteristics and smoking behaviour in the household were assessed by questionnaires during face-to-face interviews. Smokers were defined as individuals who smoked at least one cigarette per day. Regarding the father’s smoking behaviour, infants were considered exposed to SHS if the father smoked at home daily or weekly, and they were considered not exposed to SHS if the father had no smoking habit or never smoked or only smoked once a month at home.

To assess cigarette smoking status and exposure, we examined: 1) whether the mother smoked, 2) whether the father smoked, 3) whether other family members smoked and lived in the same house, 4) number of family members who smoked, 5) number of cigarettes smoked by family members, 6) number of cigarettes smoked by the father, 7) number of cigarettes smoked by the mother, and 8) frequency of father smoking at home. The socioeconomic characteristics of the parents included: marital status; the educational level of the mother and father, whether elementary (low), high school (middle), or undergraduate and higher (high); family income, whether below or on/above the minimum wage (based on Jakarta Province 2018 minimum wage); the father’s and mother’s employment status; and number of children.

This study was approved by the ethics committee of the Faculty of Medicine, Universitas Indonesia, with the code of ethics No: 895/UN2.F1/ETIK/2015.

### Statistical analysis

Descriptive statistics were used. A chi-squared test was used to compare the socioeconomic characteristics and household smoking behaviour of fathers who were smokers and those who were non-smokers and the relationship of infants’ SHS exposure and paternal smoking. The factors related to paternal smoking and SHS exposure in infants were evaluated using a multivariate logistic regression analysis.

## RESULTS

### Smoking behaviour in the household

Overall, the prevalence of smokers in the household was 71.2%, with fathers being the greatest contributors (59.6%). Maternal smoking was only 2.6% and smoking among other family members was 29.5% (Table 1). Together, the fathers and other household members smoked between 5–14 cigarettes per day, on average.

**Table 1:** Percentage of smoking fathers, mothers and other household members according to daily cigarette consumption, Jakarta, Indonesia, 2017–2019 (N=156)

*Household members*	*Non-smokers*		*Cigarette consumption (cigarettes/day)*					

			*1–4*		*5–14*		*≥15*	

	*n*	*%*	*n*	*%*	*n*	*%*	*n*	*%*
Fathers	63	40.4	16	10.3	65	41.7	12	7.7
Mothers	153	98.1	2	1.3	1	0.6	0	0
Other	110	70.5	20	12.8	23	14.7	3	1.9

### Socioeconomic characteristics, smoking behaviour in the family and smoking status of fathers

Participants were all married, with many having two (35.9%) and three or more (35.3%) children. More than half (54.4%) of the participants had family incomes under the regional minimum wage, and most mothers were unemployed (61.5%). Most of the fathers and mothers had middle-level education (75.6% and 71.8%, respectively). One-third of the children (32.7%) were exposed to paternal smoking at home, and 10.3% lived with two or more other household members who smoked.

In Table 2, the socioeconomic characteristics and smoking behaviour in the family with fathers who were smoking and not smoking were compared. Significant relationships between the smoking status of the father and the number of children and the father’s education and number of other household members who smoked were found in the bivariate analysis. Paternal smoking was more prevalent among poor families (with income under the minimum wage); however, this association was not significant. The relationships among the number of children, mother’s employment status, mother’s and father’s educational levels, other household members’ smoking status and number of other household members who smoked are shown in Table 3. Regarding the smoking status of the fathers, SHS exposure among children was likely to occur in poorer families, although the differences were not statistically significant in the bivariate analyses.

**Table 2:** Father’s smoking status in terms of socioeconomic characteristics and smoking in household, Jakarta, Indonesia, 2017–2019 (N=156)

*Characteristics*	*Categories*	*Father’s smoking status*				

		*Non-smoker (N=63)*		*Smoker (N=93)*		p*

		*n*	*%*	*n*	*%*	
**Number of children**	Infant with no siblings	25	55.6	20	44.4	0.044
	Infant with 1 sibling	18	32.1	38	67.9	
	Infant with ≥2 siblings	20	36.4	35	63.6	
**Marital status**	Married	63	40.4	93	59.6	-
**Mother’s occupation**	Unemployed	36	37.5	60	62.5	0.323
	Employed	23	48.9	24	51.1	
	Self-employed	5	35.7	9	64.3	
**Father’s occupation**	Employed	58	40.8	84	59.2	0.93
	Self-employed	9	64.3	5	35.7	
**Mother’s educational level**	Low	1	14.3	6	85.7	0.253
	Middle	45	40.2	67	59.8	
	High	17	45.9	20	54.1	
**Father’s educational level**	Low	1	25	3	75	0.044
	Middle	42	35.6	76	64.4	
	High	20	58.8	14	41.2	
**Family income status**	< Regional minimum wage	28	32.9	57	67.1	0.056
	≥ Regional minimum wage	35	49.3	36	50.7	
**Mother’s smoking status**	Non-smoker	63	41.1	89	58.6	0.148
	Smoker	0	0	4	100	
**Other household members’ smoking status**	Non-smoker	44	40.4	65	59.6	1
	Smoker	18	39.1	28	60.9	
**Number of other household members who smoke**	0	45	40.9	65	59.1	0.026
	1	16	53.3	14	46.7	
	≥2	2	12.5	14	87.5	

* Chi-squared test, significant at p<0.05.

**Table 3:** Children’s SHS exposure considering father smoking at home by socioeconomic characteristics and household smoking, Jakarta, Indonesia, 2017–2019 (N=156)

*Characteristics*	*Categories*	*SHS exposure*				

		*Not exposed (N=105)*		*Exposed (N=51)*		p*

		*n*	*%*	*n*	*%*	
**Number of children**	Infant with no siblings	37	82.2	8	17.8	0.036
	Infant with 1 sibling	33	58.9	23	41.1	
	Infant with ≥2 siblings	35	63.6	20	36.4	
**Marital status**	Married	105	67.3	51	32.7	-
**Mother’s occupation**	Unemployed	60	62.5	36	37.5	0.009
	Employed	39	83	8	17	
	Self-employed	6	46.2	7	63.8	
**Father’ s occupation**	Employed	96	67.6	46	32.4	0.773
	Self-employed	9	64.3	5	35.7	
**Mother’s educational level**	Low	2	28.6	5	71.4	0.06
	Middle	75	67	37	33	
	High	28	75.7	9	24.3	
**Father’s educational level**	Low	1	25	3	75	0.009
	Middle	75	63.6	43	36.4	
	High	29	85.3	5	14.7	
**Family income status**	< Regional minimum wage	52	61.2	33	38.8	0.094
	≥ Regional minimum wage	53	74.5	18	25.4	
**Mother’s smoking status**	Non-smoker	104	68.4	48	31.6	0.103
	Smoker	1	25	3	75	
**Other household members’ smoking status**	Non-smoker	79	72.5	30	27.5	0.045
	Smoker	25	54.3	21	45.7	
**Number of other household members who smoke**	0	80	72.7	30	27.3	0.017
	1	19	63.3	11	36.7	
	≥2	6	37.5	10	62.5	

* Chi-squared test, significant at p<0.05.

### Factors related to paternal smoking

To evaluate factors related to the smoking status of fathers, we examined the number of children, father’s educational level, family income, and number of household members who smoked in the logistic regression model. The multivariate logistic regression model showed that a larger number of children in the family increased the odds of fathers smoking (p=0.044), as fathers who had two children were more likely to smoke compared with those who had one child (OR=2.41; 95% CI: 1.02–5.67). Moreover, fathers with secondary education smoked more than those with higher education (OR=2.59; 95% CI: 1.19–5.63) (Table 4).

**Table 4:** Multivariate logistic regression analysis for factors related to father smoking, Jakarta, Indonesia, 2017–2019 (N=156)

*Factors*	*Categories*	*Father smoking*		p*

		*OR*	*95% CI*	
**Number of children**	Infant with no siblings	1	-	-
	Infant with 1 sibling	2.41	1.02–5.67	0.044
	Infant with ≥2 siblings	1.9	0.82–4.44	0.135
**Father’s educational level**	Low	4.29	0.4–45.57	0.195
	Middle	2.59	1.19–5.63	0.045
	High	1	-	-
**Family income status**	< Regional minimum wage	1.49	0.67–3.35	0.327
	≥ Regional minimum wage	1	-	-
**Number of other household members who smoke**	0	1	-	-
	1	0.55	0.24–1.29	0.171
	≥2	4.59	0.96–21.99	0.057

* Significant at p<0.05.

### Factors related to SHS exposure among infants

In the multivariate analysis (Table 5), children with one sibling (OR=3.09; 95% CI: 1.15–8.32) or who had fathers with low educational levels (OR=18.73; 95% CI: 1.54–227.93) and had two or more other household members who smoked (OR=4.54; 95% CI: 1.42–14.48) were more likely to be exposed to cigarette smoke at home from father’s smoking.

**Table 5:** Multivariate logistic regression analysis for factors related to SHS exposure among infants, Jakarta, Indonesia, 2017-2019 (N=156)

*Factors*	*Categories*	*Father smoking*		P*

		*OR*	*95% CI*	
**Number of children**	Infant with no siblings	1	-	-
	Infant with 1 sibling	3.09	1.15–8.32	0.026
	Infant with >2 sibling	2.53	0.93–6.94	0.071
**Mother’s occupation**	Unemployed	1	-	-
	Employed	0.52	0.2–1.35	0.181
	Self-employed	1.5	0.42–5.42	0.526
**Mother’s educational level**	Low	2.87	0.38–22.04	0.309
	Middle	0.79	0.28–2.22	0.650
	High	1	-	-
**Father’s educational level**	Low	18.73	1.54–227.93	0.022
	Middle	2.66	0.92–7.65	0.07
	High	1	-	-
**Family income status minimum wage**	< Regional minimum wage	0.98	0.38–2.5	0.962
	≥ Regional minimum wage	1	-	-
	**Mother’s smoking status**	Non–smoker	1	-	-
Smoker		4.78	0.46–49.33	0.19
**Number of other household members who smoke**	0	1	-	-
	1	1.55	0.63–3.8	0.341
	≥2	4.54	1.42–14.48	0.011

* Significant at p<0.05.

## DISCUSSION

The prevalence of household smokers in this study was quite high, and it was primarily attributed to paternal smoking. The results of this study are in line with the study of Best et al.^22^ who examined 438336 households in rural areas in Indonesia where paternal smoking reached 73.7%. The study of Semba et al.^21^ showed that in 175583 urban poor households in Indonesia in 2007, the prevalence of paternal smoking reached 73.8%.

This study showed that lower levels of education were associated with paternal smoking and SHS exposure among infants. Several studies showed that parents’ low educational level was associated with SHS exposure in children^15,21–25^. In this study, a significant relationship was found between the number of family members who smoked and exposure to cigarette smoking among infants, and there was a tendency that the greater the number of family members who smoked, the greater the risk of fathers becoming smokers. This occurred if the baby’s parents lived at home with their extended families. These results are in line with the study by Mwaniki and Gray^26^. They are also consistent with a meta-analysis study by Leonardi-Bee et al.^27^ and systematic literature review by Kusel et al.^18^, which found that the use of cigarettes significantly increased if at least one parent smoked, if mothers smoked, fathers smoked, both parents smoked, siblings smoked and other family members smoked. In another study, tobacco use was influenced by the number of close friends who smoked^28^.

In the present study, we found that a large number of children in the family was associated with higher odds of smoking in fathers and SHS exposure in children. Hawkins and Berkman^29^ also found that babies with siblings were at greater risk of SHS exposure. This study suggests that parents in Indonesia should change their smoking behaviours and rules at home, especially if they have more children. Further studies are needed to better understand these decision-making processes and help recognise critical periods for appropriate interventions^29^. Moreover, health promotion programmes should place more stress on tackling smoking in fathers who have infants with one or more siblings. Indeed, fathers’ and other household members’ smoking behaviours have received far less attention or health promotion consideration than mothers’ smoking behaviours. Protecting babies from smoking parents and other smokers in the family is crucial in diminishing SHS exposure in early infancy, when the risks of asthma attacks, respiratory infections, ear infections, and SIDS are more common^30,31^.

In this study, no relationship was found between family income and father’s smoking status. However, higher percentages of paternal smoking and SHS exposure among infants occurred in low-income families than in high-income families. Previous studies showed that lower social class increased the risk of exposure to cigarette smoke in women and children at home^23,25,32^. Furthermore, paternal smoking was associated with an increased risk of stunting and wasting in children from urban poor families^21^, and underweight and stunting in rural children in Indonesia^22^. According to Semba et al.^21^, the risk of undernutrition in early childhood was due to the fact that a large proportion of family income was used for cigarettes and a small proportion for buying food.

Other than the risk of undernutrition, the high prevalence of smoking in households and exposure to SHS in infants, the current study raises concerns about the risk of cardiovascular diseases (CVDs) at a younger age in Indonesia. Forty-five per cent of CVD deaths in those aged 30–44 years were more likely to be caused by tobacco use. CVDs are the number one cause of death with 558736 each year (36.3% of all deaths). CVD deaths caused by tobacco usage were 147510 or 26% of all CVD deaths each year^3^. Therefore, it is crucial to control tobacco to prevent and control disability and death caused by CVDs.

Getting smokers to quit would be problematic as smoking is part of the Indonesian culture. To address this, the most effective approach is indirectly asking whether father’s responsibility for their partners and children is of larger cultural value^33^. Regarding this, community-based smoke-free home initiatives in Yogyakarta, Indonesia, could redefine smoking cessation as a health issue for women and children^34^. This policy will go hand-in-hand with government regulations prohibiting smoking on public transport and public places, and providing smoking cessation services in hospitals and health centres etc.

### Limitations

A limitation of this study was the small sample size, which led in some cases to wide confidence intervals. Further, we used a questionnaire to measure self-reported SHS and did not objectively measure nicotine or cotinine levels. However, several studies that have relied on self-reports on smoking behaviours found good agreement between cotinine/nicotine levels and self-reports, indicating the suitability of questionnaires as a means of measuring smoking behaviour and exposure^35–37^. The current study also did not include e-cigarettes or heat-not-burn tobacco users in Indonesia. These limitations will need to be addressed in future studies.

## CONCLUSIONS

The findings of the present study highlight that infants who are only a few months old and living with smokers are exposed to SHS at home. The father’s educational level, infants having siblings, and/or the number of other household members who smoke affect father’s smoking status and SHS exposure among infants at home. Designing an appropriate anti-smoking education program to target families is recommended. Furthermore, comprehensive tobacco control programmes to increase the adoption of smoke-free homes are likely to be an effective way to reduce SHS exposure and promote decreased cigarette smoking in families with children.
